# Renal cell carcinoma with intracardiac tumor thrombus extension: Radical surgery yields 2 years of postoperative survival in a single-center study over a period of 30 years

**DOI:** 10.3389/fonc.2023.1137804

**Published:** 2023-02-01

**Authors:** Pavel Zacek, Milos Brodak, Jan Gofus, Jan Dominik, Petr Moravek, Miroslav Louda, Miroslav Podhola, Jan Vojacek

**Affiliations:** ^1^ Department of Cardiac Surgery, Charles University, Faculty of Medicine in Hradec Kralove and University Hospital in Hradec, Kralove, Czechia; ^2^ Department of Urology, Charles University, Faculty of Medicine in Hradec Kralove and University Hospital in Hradec, Kralove, Czechia; ^3^ The Fingerland Department of Pathology, Charles University, Faculty of Medicine in Hradec Kralove and University Hospital in Hradec, Kralove, Czechia

**Keywords:** renal cell carcinoma, radical surgery, deep hypothermic circulatory arrest, cardiopulmonary bypass, intracardiac tumor thrombus extension

## Abstract

**Background:**

Renal cell carcinoma (RCC) with tumor thrombus extension into the right atrium (level IV) is a rare life-threatening clinical condition that can only be managed by means of a combined urological and cardiac surgical approach. The early and late outcomes of this radical treatment were analyzed in a large single-institution series over a period of 30 years.

**Methods:**

In 37 patients with RCC and intracardiac tumor thrombus extension, nephrectomy was performed followed by the extraction of the intracaval and intracardiac tumor thrombus under direct visual control during deep hypothermic circulatory arrest (DHCA). Recently, in 13 patients, selective aortic arch perfusion (SAAP) was instituted during DHCA.

**Results:**

In all patients, precise removal of the tumor thrombus was accomplished in a bloodless field. The mean duration of isolated DHCA was 15 ± 6 min, and 31.5 ± 10.2 min in the case of DHCA + SAAP, at a mean hypothermia of 22.7 ± 4°C. In-hospital mortality was 7.9% (3 patients). In Kaplan–Meier analysis, the estimated median survival was 26.4 months whereas the 5-year cancer-related survival rate was 51%.

**Conclusions:**

Despite its complexity, this extensive procedure can be performed safely with a generally uneventful postoperative course. The use of cardiopulmonary bypass with DHCA, with the advantage of SAAP, allows for a safe, precise, and complete extirpation of intracaval and intracardiac tumor mass. Late outcomes after radical surgical treatment in patients with RCC and tumor thrombus reaching up in the right atrium in our series justify this extensive procedure.

## 1 Introduction

Renal cell carcinoma (RCC) with intracardiac tumor thrombus extension is a serious clinical finding that can be ultimately cured only by means of a radical surgery. Since 1984, when surgical treatment was described for the first time by Marshall with use of deep hypothermic circulatory arrest (DHCA) (1), it has been clearly evident that the accomplishment of the operation is a challenging surgical task. Conceptually, two conflicting objectives need to be orchestrated: a precise and complete removal of tumor mass including the kidney and voluminous tumor thrombus located in the central venous vasculature and heart chambers while simultaneously reducing the complexity of the procedure and its burden for the patient. The use of DHCA serves well the purpose of exact tumor removal, but its complexity has provoked many authors to suggest less extensive surgical techniques while maintaining a comparable control over tumor extraction.

Four decades after, three main questions persist that include bold decisions for major surgery in a given patient, optimal technical management of the operation, and, finally, defining a rewarding outcome of the radical approach. A single-center experience comprising 37 patients over a period of 30 years, from Czech Republic, a country with the highest incidence of RCC, is to be presented.

## 2 Materials and methods

Over the period of 30 years (from January 1993 to September 2022), 3,015 patients were operated for RCC at our tertiary care center. Nephrectomy was performed in 2,255 patients, renal resection in 1,021 patients, and nephrectomy with the extirpation of tumor thrombus in 388 patients (12.9%), respectively.

Among the subgroup requiring tumor thrombus extirpation, 37 patients (1.2%) had the tumor thrombus extending into the right atrium [level IV according to Neves (2)]. There were 22 men, with a median age of 63 years (range 42–80 years) and 15 women, with a median age of 60 years (range 35–75 years). Aside from patient #1 (operated in 1978, giving a time span of literally 44 years), all others have been operated since 1993 at a yearly rate of one-to-three cases. Patient demographic data are presented in **Table 1**. The data were obtained retrospectively from in-hospital patient records and outpatient department visits. The follow-up was 100% complete. Patient-informed consent was waived. The study was approved by the local ethics committee. The complete dataset may be shared upon reasonable request to the first or corresponding author.

**Table 1:** The patient demographic characteristics and perioperative data.

	Patient cohort (n = 37)
Female sex, n (%)	15 (41%)
Age [years], median (IQR)	63 (42–80)
BMI [kg/m^2^], median (IQR)	25.7 (18.4–40.9)
Diabetes, n (%)	6 (16%)
Hypertension, n (%)	8 (22%)
Coronary artery disease, n (%)	4 (11%)
Chronic obstructive pulmonary disease, n (%)	0
Entry creatinine [mmol. L ^-1^], ø ± SD	115 ± 30
Discharge creatinine [mmol. L ^-1^], ø ± SD	137 ± 30
Procedural time [min], ø ± SD	437 ± 113
Hypothermia DHCA alone [°C], ø ± SD	20.4 ± 2.8
Hypothermia DHCA + SAAP [°C], ø ± SD	24.6 ± 1.5
Circulatory arrest DHCA alone [min], ø ± SD	16 ± 6
Circulatory arrest DHCA + SAAP [min], ø ± SD	31 ± 10
Postoperative blood loss [ml], median (IQR)	525 (150–3,600)
Duration of intubation [h], median (IQR)	21 (5–96)
Concomitant procedures (n):	
*Coronary artery bypass grafting*	2
* Cholecystectomy*	2
* Splenectomy*	1
* Aortic valve replacement*	2
* Pulmonary embolectomy*	1

BMI, body mass index; DHCA, deep hypothermic circulatory arrest; IQR, interquartile range; SAAP, selective aortic arch perfusion; SD, standard difference.

The diagnosis of the tumor, its extent, and staging were confirmed by abdominal sonography; computed tomography with angiography; echocardiography; and, eventually, magnetic resonance imaging, chest X-ray, skeletal scintigraphy, or pulmonary and brain-computed tomography.

### 2.1 Indication for surgery

Indication in all patients was weighed against the risks of extensive surgery. An oncological contraindication was the presence of multiple metastases. Eventual solitary metastasis amenable to later treatment would not be a contraindication. However, no such patient exhibited signs of it in our series. Further risk assessment was based on an approach similar to a conventional cardiac surgery. Left ventricular performance, valvular morphology, and coronary arteries were routinely examined. Easily repairable cardiac disorders (valve replacement and coronary revascularization) were no contraindication for surgery and were performed concomitantly. Soft criteria (such as frailty) were taken into consideration. In general, the patients presented with very little symptomatology. Surgery, even despite advanced age in some cases (two octogenarians were a part of the study cohort), was offered to all referred but one patient with terminal hepatic failure.

### 2.2 Surgical technique

**Figure 1:**
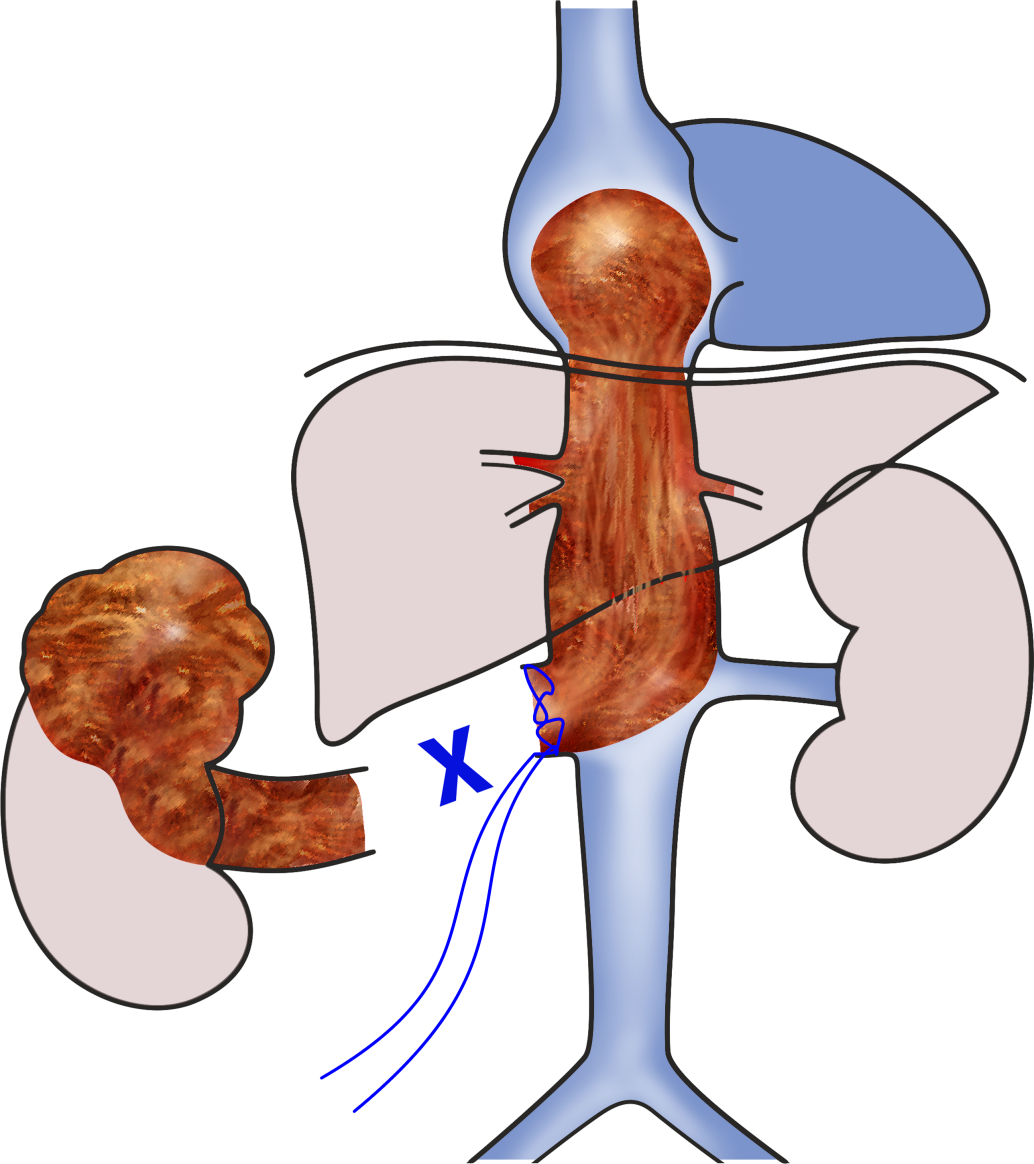
Scheme of the 1st phase of the surgery. The right kidney with a tumor was already removed, and level IV intracardiac tumor thrombus was secured in place by suturing the stump of the right renal vein.

As described elsewhere (3), the procedure was performed in cooperation of urology and cardiac surgery teams. Briefly, a radical nephrectomy was performed first by the urology team *via* a chevron incision for a left-sided tumor or a liberal subcostal incision for right-sided renal carcinoma. After nephrectomy, the renal vein stump containing the tumor thrombus was provisionally secured with a suture ligature to prevent thrombus migration (**Figure 1**).

Second, the cardiac surgery team performed the median sternotomy. On full heparinization, the ascending aorta was cannulated for the arterial line of the cardiopulmonary bypass (CPB) in a standard fashion. For venous drainage, a selective cannulation of the superior vena cava and right atrium (a shallow insertion of the cannula in the direction of the inferior vena cava) was performed and the CPB was initiated. The patient was cooled down to deep hypothermia.

In an earlier portion of the series (patient 1–19), the target core temperature was 20°C (urinary bladder and rectal temperature measurement) for patients with the thrombus obstructing the dilated inferior vena cava without any signs of circumfluence. In patients with preserved circumfluence and easy removal expectation, the cooling was stopped earlier.

**Figure 2:**
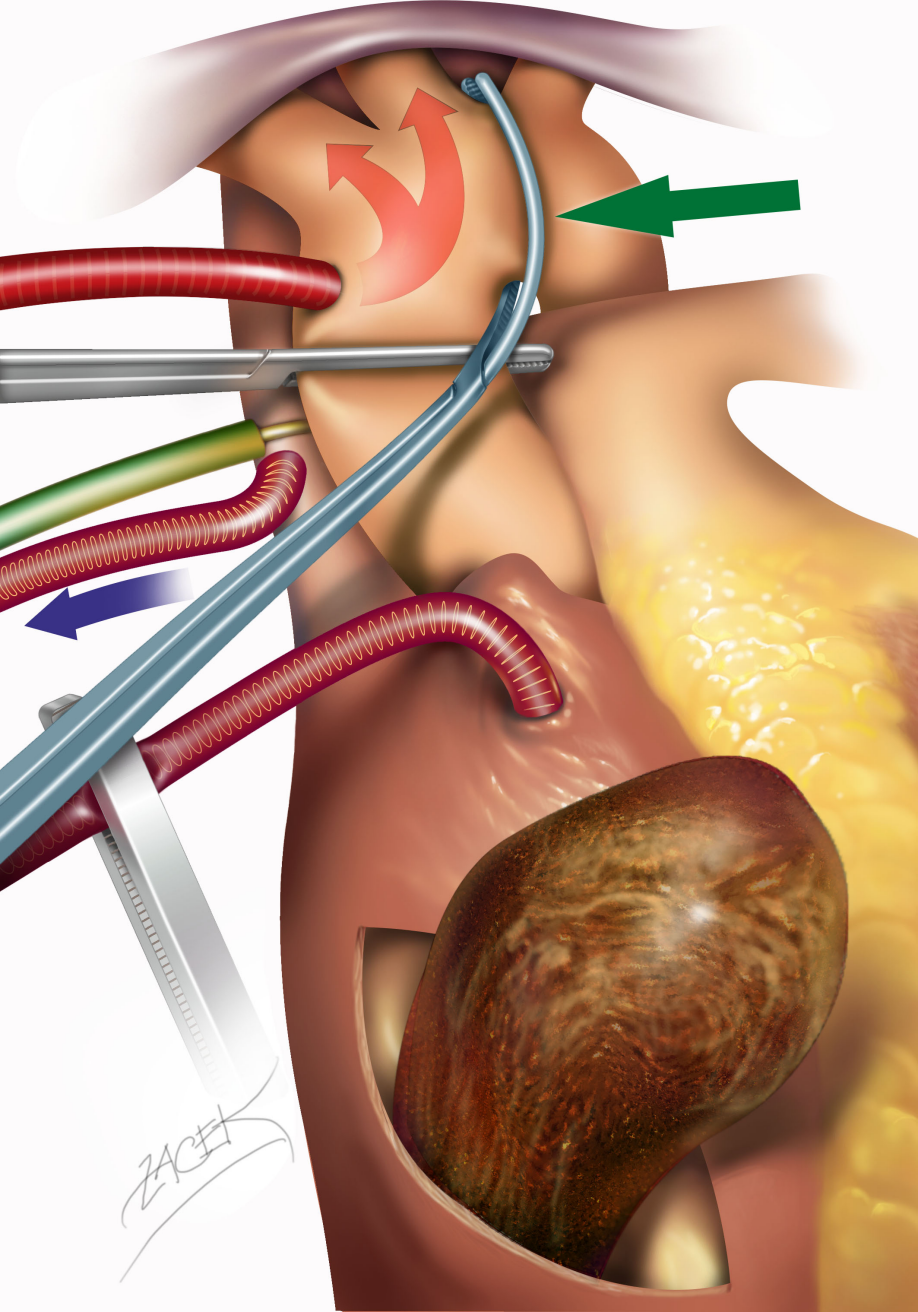
Accomplishment of selective aortic arch perfusion. After standard aortic cannulation and cross-clamping, a second curved aortic clamp (green arrow) is applied obliquely beyond the origin of the left carotid artery. In this manner, the aortic cannula perfuses during deep hypothermic circulatory arrest, predominantly the head arteries, at a reduced rate and under near infrared spectroscopy NIRS cerebral oximetry monitoring.

For greater flexibility and safety of DHCA, we proposed the technique of selective aortic arch perfusion (SAAP) in 2014 (4) and used it in the latter 13 patients. Briefly, before the institution of CPB, the ascending aorta and proximal aortic arch were gently mobilized to allow for the placement of a second aortic clamp obliquely beyond the left carotid artery (**Supplementary Video 1**). After standard aortic cross-clamping and cardioplegic cardiac arrest, the second cross-clamp was applied and selective perfusion of the aortic arch was started at 1–1.5 L.min^-1^ under near-infrared spectroscopy cerebral oximetry monitoring (FORE-SIGHT, Casmed, CT, USA). This technique enabled a liberal safe duration of circulatory arrest at less profound hypothermia (25°C, see **Figure 2**).

**Figure 3:**
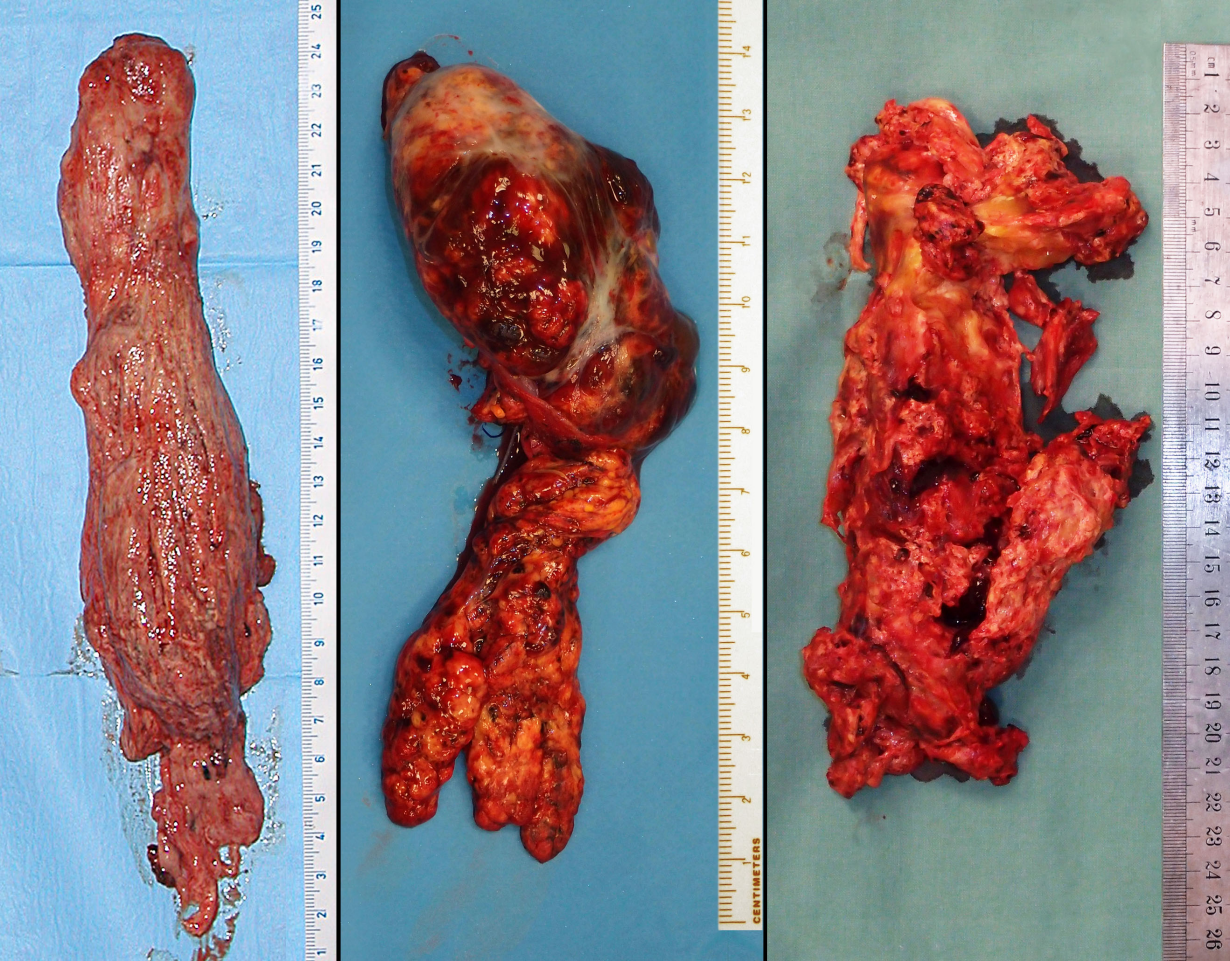
Different types of tumor thrombus solidity, varying from a relatively compact to very fragile and necrotizing mass.

At the desired temperature, the cardioplegic cardiac arrest and circulatory arrest were instituted. A short transverse right atriotomy was performed close to the junction with the inferior vena cava to obtain control over the intracardiac end of the tumor thrombus. Simultaneously, a tear-shaped abdominal cavotomy was performed circumcising the renal vein stump and extended cranially. According to its solidity, the tumor was extracted both by gentle pulling and blunt or sharp dissection. A difficult area used to be its intrahepatic course with junction of the hepatic veins. A meticulous search for any tumor remnants was mandatory (contralateral renal vein, distal vena cava, right atrium, and ventricle). While most tumor thrombi could be extracted relatively easily, some adhered firmly and required a piecemeal extirpation (see **Figure 3**).

The cavotomy and atriotomy were closed with running suture. Eventual concomitant cardiac surgery was performed in the rewarming phase. After rewarming, CPB was weaned off, perfusion cannulas were removed, and both sternotomy and laparotomy were closed in a standard manner. A postoperative course was led in a standard fashion. After discharge, the patients were regularly followed up by an oncologist and urologist/cardiologist, as necessary.

### 2.3 Statistical analysis

For recorded data, means or medians were calculated according to value distribution. Standard deviation and the interquartile range were used for the statement of the associated uncertainty. The postoperative overall and cancer-specific survival were estimated using the Kaplan–Meier method. Statistical analysis was performed using software NCSS 2007 (NCSS, Kaysville, UT, USA).

## 3 Results

A total of 38 complex procedures were performed in 37 patients (one patient required a repeated procedure for isolated local recurrence of intracaval tumor thrombus 9 months after the index procedure). In all cases, the procedure was accomplished successfully under direct visual control. The head of the tumor mass usually filled the caudal third of the right atrium; in one case, a fast progression of the tumor into the right ventricle during the last 10 days was noticed. A large bulky head necessitated the transection of the tumor to allow the passage of the rest of the tumor caudally to abdominal cavotomy in three patients. Due to the variable composition and adherence of the tumor thrombus, the required duration of circulatory arrest varied from 5 to 33 min (median 16 min) in the DHCA alone series, and from 10 to 45 (median 28 min) when DHCA + SAAP was used. From 13 patients with DHCA + SAAP, in 2, the second clamp had to be placed beyond the brachiocephalic trunk due to the calcifications of the left carotid resulting in unilateral brain perfusion during SAAP. No side differences in cerebral perfusion were noticed. In two patients, hypothermia had to be avoided due to the presence of cold agglutinins; a non-obstructive tumor thrombus was removed in normothermia and low-flow periods of 23 and 25 min. In eight patients, several concomitant procedures were performed: aortic valve replacement, coronary artery bypass grafting, cholecystectomy, splenectomy, pulmonary embolectomy, and others (**Table 1**).

There were three early postoperative deaths (in-hospital mortality 7.9%). Two of them were due to multiorgan failure early in the series (on postoperative day 10 and 32); the third was a stroke in a patient with DHCA alone (on postoperative day 7). In all other patients, the postoperative course was relatively uneventful with regard to the considerable length of the surgery (a median of 6.8 h).

Histologically, a clear cell carcinoma was found in 34 cases (89%) and papillary renal carcinoma in three cases. Fuhrman grade was classified to be 3 in 28 of 30 cases; novel modified classification was applied in last seven patients (5) (**Table 2**).

**Table 2:** Histopathologic tumor characteristics.

	Study cohort (n = 38)	
Right-sided tumor, n (%)	19 (50%)	
Left-sided tumor, n (%)	19 (50%)	
Histology, n (%):		
* Clear cell carcinoma*	34 (89%)	
* papillary carcinoma*	3 (8%)	
* Unclassified carcinoma*	1 (3%)	
Fuhrmann grade (in 31 patients), n (%):		
* Grade 3*	30 (97%)	
* Grade 4*	1 (3%)	
Modified grading (5) (in the last 7 patients), n (%):		
* Grade 2*	2 (29%)	
* Grade 3*	2 (29%)	
* Grade 4*	3 (43%)	
TNM classification (n, %):		
* T3b*	9 (24%)	
* T3c*	27 (71%)	
* N0*	9 (24%)	
* N1*	5 (13%)	
* N2*	1 (3%)	
* NX*	23 (61%)	
* M0*	38 (100%)	

**Figure 4:**
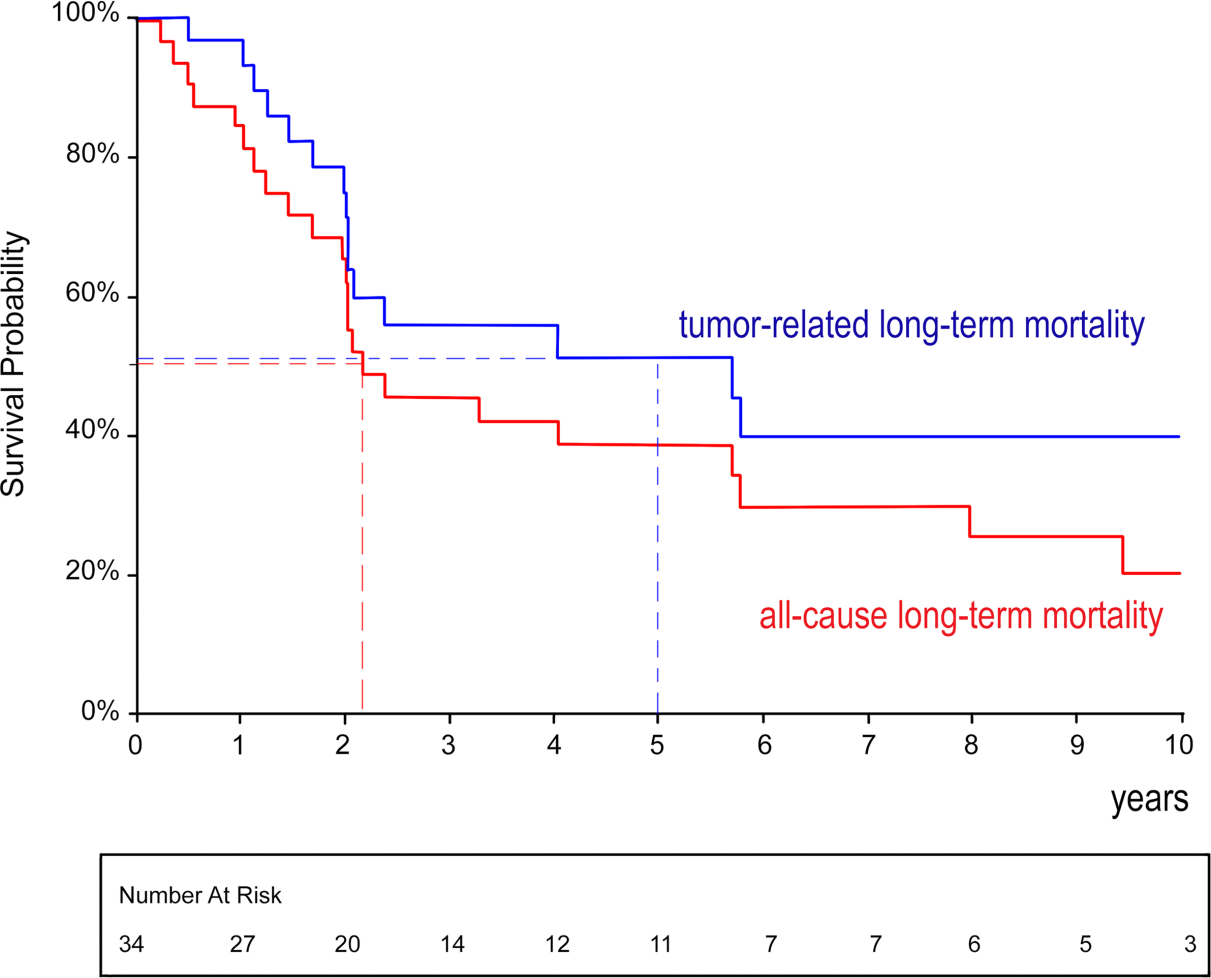
Kaplan–Meier analysis of overall and cancer-related survival after radical surgery for renal cell carcinoma with level IV tumor thrombus.

A total of 34 patients entered the follow-up (135 patient/years cumulatively). There were 16 deaths attributed to metastatic cancer recurrence; 7 deaths were caused by other reasons (stroke, myocardial infarction, acute pancreatitis, etc.). The long-term survival was analyzed in a Kaplan–Meier plot with regard to all-cause and cancer-related mortality (see **Figure 4**). Generally, the median estimated overall survival was 2.2 years; the 95% confidence limits (CLs) were 2.0–5 years. The median estimated cancer-related survival was 5.7 years (95% CLs 2.0–5.8 years). The 5-year estimated cancer-related survival was 51%. There were 39% of patients who may benefit from long-term cancer-free survival.

## 4 Discussion

RCC with a true level IV [i.e., intracardiac according to Neves (2)] tumor thrombus extension is a complex and serious clinical condition that combines advanced oncological involvement with a massive obstruction in central venous circulation. This, together with its very rare occurrence, imposes challenging and controversial questions on the strategy of surgery, which, anyway, remains the only radical treatment. Simply said, the necessity of intracardiac tumor removal increases considerably the complexity of procedure and, therefore, every effort to alleviate the burden of surgery has been justified. On the other hand, all techniques with only indirect control of tumor extirpation carry the risk of removal incompleteness and massive pulmonary embolism.

The key conundrum is the morphological properties of the tumor thrombus—its shape, consistency, and strength of adhesion to the caval wall. All three qualities significantly influence the ease or difficulty of tumor removal, and none of the latter two can be judged preoperatively in other than the only indirect way. Basically, two types of tumor thrombi can be found. The first one, encountered in approximately two-thirds of instances, has a relatively solid composition and smooth surface covered with a quasi-capsule. Such tumor can be separated from the caval wall with blunt finger dissection, and only few fibrous attachments to the orifices of intrahepatic veins have to be cut. Removal is technically relatively easy by pulling the tumor caudally with the exception of a dumbbell-shaped thrombus where the voluminous intraatrial portion has to be transected and evacuated *via* the right atrium and the rest of the tumor pulled down through the cavotomy. The second type, contrariwise, has inconsistent, necrotizing, and friable structure parts that firmly adhere to the caval wall, while others disintegrate into fragments even at minimal manipulation. The risk of embolization is high, and a meticulous search inside the caval and atrial lumen under direct vision is mandatory to avoid this.

Very few indirect signs may predict the complexity of the finding. On computed tomography, larger tumor dimensions herald greater obstruction and heavier adhesions. Inhomogeneous density of the tumor also foretells a fragile composition of the mass. Echocardiography may reveal slight tumor rocking and the traces of circumferential flow that both prognosticate easier removal. The tip of the tumor thrombus is usually a bulky rounded head moving inside the right atrium, but a snake-like tail rapidly growing into the right ventricle within days was also encountered. The final assessment of the tumor properties, however, can be made only after cavotomy.

Stimulated by experiencing sometimes surprisingly difficult removal, we operate on all patients with intracardiac extension of renal cell carcinoma with the use of CPB and DHCA. In 2014, we described the use of a second aortic cross-clamp to start SAAP during circulatory arrest and this maneuver became our standard technique. It enables us to safely extend the duration of circulatory arrest beyond critical temperature limits and reduce the degree of cooling. Under these conditions, the removal of tumor thrombus masses can be accomplished very safely and precisely in a bloodless field during a stress-free time interval no matter how difficult the extirpation may happen to be. Moreover, based on accumulated experience with this strategy and with meticulous attention to hemostasis, the postoperative courses are surprisingly smooth and uneventful even despite the overall length of the operation.

The use of CPB + DHCA has long paved the way (6–9). Simultaneously various pathways to avoid it for the sake of lesser complexity of the procedure have been attempted (10). Generally, should there be a trade-off for a complete avoidance of extracorporeal circulation and hypothermia, it means having only an external control over manipulation with the tumor thrombus (pushing the tumor head through the atrial wall, suprahepatic caval compression, blind milking, etc.) (11). With all due respect to the benefits of a faster and less complex procedure, in some tumors, its dimensions and fragmentation may be hazardous for incomplete removal and pulmonary embolization (12, 13). Later, the liver transplant techniques were successfully used for obtaining access to the retrohepatic vena cava by complete liver mobilization and piggyback liver dislocation (14). Excellent exposure is obtained; the intracardiac thrombus control still remains only indirect *via* diaphragm incision, and hepatic ischemia in normothermia is of concern (15). Transesophageal echocardiographic guidance over the tumor extraction through cavotomy without CPB is helpful in detecting residual thrombus, yet it does not prevent fearsome thrombus migration and embolization (16). In his systematic review of the literature, Gaudino concludes the use of CPB + DHCA to be the preferred strategy for its defined advantages (17). Recently, the emerging robotic technique was successfully used for nephrectomy and tumor thrombus removal (level II–III and, recently, intracardiac level IV) in CPB+/-DHCA, although it was admitted to be still rather complex; a robot-controlled endoluminal venacavoscopy may feature additional technical benefit (18–20).

Long-term survival remains the crucial issue that depends mostly on complete removal of the kidney tumor, including the entire tumor thrombus from the inferior vena cava and right atrium (21). Our data show that the survival has remained generally unchanged over the years (median slightly above 2 years) since the initial decrease of survival is dictated by the oncological aggressiveness of the disease. Later, the curve flattens yielding the probability of 5-year cancer-related survival to be approximately 50%, and 40% of patients are to be cured for even longer. Apart from surgical treatment, our therapeutic options are very limited. Chemotherapy and radiotherapy are almost ineffective (22). Biological treatment with checkpoint inhibitors, which is currently indicated for metastatic RCC, has shown a certain effectiveness (23). For a relatively small number of these patients, adjuvant or neoadjuvant administration of biological treatment is not clinically evaluated and recommended. Currently, clinical trials evaluating adjuvant administration in high-risk RCC are underway, and the preliminary results look promising (24, 25). If proven to be effective, it would be of great benefit in this group of patients with high-risk kidney cancer.

### 4.1 Limitations

Due to the very rare occurrence of a reported clinical finding, this is a retrospective observational study based on slow-growing series of patients treated over a considerable time span. Although the main principles of the surgery remained unchanged, various substantial advances in perioperative care have been implemented over this period and contributed to the easier patient’s tolerance of this extensive procedure in the recent era.

## 5 Conclusion

RCC with extensive intracardiac tumor thrombus can be treated surgically in a standardized manner as presented in our single-institution series. The combined approach of urologists and cardiac surgeons with the use of CPB and DHCA enables a safe and predictable accomplishment of the complex procedure. Selective perfusion of the aortic arch during DHCA provides standardized safe conditions for precise thrombus removal even in very complicated findings. With a meticulous hemostatic surgical technique, the role of hypothermia itself does not seem to have a negative impact on the postoperative course. The long-term general and cancer-related survival do justify offering this extensive radical surgery in dedicated centers to patients with absent metastases and a reasonable biological and cardiovascular status.

## Data availability statement

The raw data supporting the conclusions of this article will be made available by the authors, without undue reservation.

## Ethics statement

The studies involving human participants were reviewed and approved by Ethics Committee, University Hospital Hradec Králové. Written informed consent for participation was not required for this study in accordance with the national legislation and the institutional requirements.

## Author contributions

PZ – substantial contribution to the concept, data collection, data analysis, illustrations, and drafting of the manuscript. MB – substantial contribution to the concept, data collection, and critical revision of the manuscript. JG – data collection, data analysis, and drafting of the manuscript. JD – substantial contribution to the concept and data collection. PM, ML, and MP – data collection. JV – critical revision of the manuscript and final approval of the version to be published. All authors contributed to the article and approved the submitted version.

## Supplementary material

Supplementary Video 1 - The operative setting for selective aortic arch perfusion. *Cardiopulmonary bypass instituted by a standard cannulation of the ascending aorta, venous cannulation of the superior vena cava, and a shallow cannulation of the right atrium. The aortic arch had been dissected free and prepared for the second clamp beyond the origin of left carotid artery—blue line.*
